# Validation of response assessment according to international consortium for MDS/MPN criteria in chronic myelomonocytic leukemia treated with hypomethylating agents

**DOI:** 10.1038/bcj.2017.41

**Published:** 2017-05-12

**Authors:** M Duchmann, T Braun, J-B Micol, U Platzbecker, S Park, S Pilorge, O Beyne-Rauzy, N Vey, M Sébert, B Gruson, P-Y Dumas, R Guieze, M-L Chretien, K Laribi, Y Chait, L Legros, L Sahnes, P Hirsch, C Salanoubat, E Solary, P Fenaux, R Itzykson

**Affiliations:** 1 Université Paris Diderot, Paris, France; 2 Service d’hématologie, Hôpital Avicenne, Université Paris XIII, Assistance Publique Hôpitaux de Paris, Bobigny, France; 3 Service d’hématologie, Gustave Roussy, Université Paris-Sud, Villejuif, France; 4 Medizinische Klinik und Poliklinik I, University Hospital Carl Gustav Carus Dresden, Dresden, Germany; 5 Clinique Universitaire d’hématologie, CHU de Grenoble Alpes, Université Grenoble Alpes, Grenoble, France; 6 Service d’hématologie, Hôpital Cochin, Université Paris V, Assistance Publique Hôpitaux de Paris, Paris, France; 7 Service de Médecine Interne, IUCT Oncopôle, Université Toulouse III, Toulouse, France; 8 Service d’hématologie, Institut Paoli-Calmettes, Université Aix-Marseille, Marseille, France; 9 Service d’hématologie Sénior, Hôpital St Louis, Université Paris Diderot, Paris, France; 10 Service d’hématologie, CHU d’Amiens, Université d’Amiens-Picardie, Amiens, France; 11 Service d’hématologie et de Thérapie Cellulaire, CHU de Bordeaux, Université Bordeaux II, Bordeaux, France; 12 Service d’hématologie, CHU Clermont-Ferrand, Université d’Auvergne, Clermont Ferrand, France; 13 Service d’hématologie, CHU François Mitterrand, Université de Bourgogne, Dijon, France; 14 Service d’hématologie, CH Le Mans, Le Mans, France; 15 Service d’hématologie, CH de Montfermeil, Montfermeil, France; 16 Service d’hématologie, CHU de Nice, Université Nice-Sophia-Antipolis, Nice, France; 17 Service d’hématologie, CH Saint Jean, Perpignan, France; 18 AP-HP, Hôpital Saint-Antoine, Service d’Hématologie clinique et de thérapie cellulaire, Paris, France; 19 Service d’hématologie, CH Sud Francilien, Corbeil Essones, France; 20 Service d’hématologie Adulte, Hôpital Saint-Louis, Université Paris Diderot, Assistance Publique Hôpitaux de Paris, Paris, France

The hypomethylating agents (HMA) azacitidine (AZA)^1, 2^ and decitabine (DAC)^3, 4^ are active in Chronic Myelomonocytic Leukemia (CMML) with overall response rates (ORR) of 40–70%, translating into median overall survivals (OS) of 12–22 months. Response to HMA in these CMML cohorts was mostly evaluated according to IWG-2006 criteria,^5^ which do not assess improvement of myeloproliferative features nor quality of life. In myelodysplastic syndroms (MDS) patients treated with HMA, these criteria have limited impact in predicting OS.^6, 7^ Normalization of WBC and monocyte counts, regression of splenomegaly or other extra-medullary disease, and improvement of quality of life have been reported in CMML patients treated with HMA^1, 8^ and studies in MDS and primary myelofibrosis have shown that symptom improvement could be correlated to a prolonged OS.^9, 10^

To capture both MDS and myeloproliferative neoplasm (MPN) features in a singly response scale in CMML, an international expert panel, the MDS/MPN International Working Group, proposed new response criteria for MDS/MPN, hereafter referred to as overlap-MDS/MPN criteria.^11^ These new criteria take in consideration bone marrow and peripheral blood blast reduction and improvement of cytopenias, but also account for correction of WBC, monocyte and peripheral immature myeloid cell counts (IMC), regression of splenomegaly and other extra-medullary disease. These criteria also assess correction of myelofibrosis, generally moderate in CMML,^12^ and propose a provisional entity of ‘clinical benefit’ solely based on improvement assessed with the MPN-SAF scoring system,^13^ which has been developed in primary myelofibrosis and has never been validated in MDS/MPN. These new criteria remain to be validated.

To validate these overlap-MDS/MPN criteria in the most frequent entity amongst MDS/MPN, namely CMML, we updated clinical data from 79 CMML patients treated by AZA or DAC included in GFM CMML clinical trials (EudraCT No. 2008-000470-21)^4^ or registry (PHRC MAD-06).^14^ The cohort included 56 males and 23 females, with a median age of 72 years. At HMA onset, 57% of patients had CMML-1 and 43% had CMML-2. Splenomegaly was present in 40% of cases. Median Hb, WBC, ANC and platelets were 9.7 g/dl, 14.5 × 10^9^/l, 7.1 × 10^9^/l and 101 × 10^9^/l, respectively. CPSS prognosis score was low in 12%, intermediate-1 in 20%, intermediate-2 in 51% and high in 17%. The GFM prognostic risk was low in 32%, intermediate in 36% and high in 32% assessable patients respectively. Forty-eight patients (61%) received AZA and 31 (39%) received DAC, with a median interval between diagnosis and HMA onset of 5 months (inter-quartile range (IQR) 1.1–26.3). The median number of cycles was 9 [IQR 5-17]. Detailed baseline characteristics of patients are provided as Supplementary Data.

Median follow-up was 59 months, during which 33 patients (42%) had AML transformation and 11 (14%) received an allogeneic stem-cell transplant (ASCT). Median OS was 27.9 months (IQR 14.7–60.6) and median AML-free survival (AMLFS) was 23.1 months (IQR 10.3–58.5). Expectedly, patients treated by DAC in the GFM trial for advanced CMML had poorer CPSS risk (*P*=0.01) and GFM risk (*P*=0.02) than AZA patients, resulting in poorer OS (median 39.8 months for AZA vs 18.4 months for DAC, *P*=0.002) and AMLFS (median 29.7 months for AZA vs 16.7 months for DAC patients, *P*=0.003). The baseline differences between these two patient populations were addressed by adjusting for HMA in all analyses.

Initial response was assessed after a median of four cycles (IQR 3–6) and is reported as Supplementary Data. Best response with IWG-2006 and overlap-MDS/MPN was achieved after a median of five cycles (IQR (4–7)), without significant delay between criteria sets (paired *t*-test *P*=0.43). According to IWG-2006, ORR was 57%. IWG-2006 responses included complete response (CR) in 20% of cases, marrow CR (mCR) with Hematological Improvement (HI) in 13%, mCR without HI in 10%, stable disease (SD) with HI in 14%. No patient achieved PR; 19% patients had SD without HI and 24% had progressive disease (PD). Regarding overlap-MDS/MPN criteria, the ORR was 71%. In our dataset, improvement of clinical symptoms was assessed retrospectively by reviewing patients’ charts, instead of applying the MPN-SAF scoring system^13^ as recommended. Similarly, we could not evaluate improvement of myelofibrosis, because the use of trephine biopsies is not part of the French guidelines for CMML. However, diffuse myelofibrosis is infrequent in CMML and can be suspected in case of dry tap. Overlap-MDS/MPN responses included CR in 13%, optimal marrow response (OMR) with clinical benefit (CB) in 18% (including CB in spleen size (CB-Spl) 6%, and CB in general symptoms (CB-Sym) 1%), OMR without CB in 15%, partial marrow response (PMR) in 1% and SD with CB in 24% (including CB-Spl 5%, and CB-Sym 1%). No patient achieved PR. Twenty-four percent of patients had SD, and 5% had progressive disease.

Overall response status at best response between IWG-2006 and overlap-MDS/MPN was concordant in 86% of cases, corresponding to a Cohen’s Kappa^15^ of.7, indicating a relatively good agreement between response criteria. Sources of discrepancies are summarized in Table 1. All responders per IWG-2006 criteria achieved some form of response with overlap-MDS/MPN criteria. Among responders with both sets of criteria, six patients achieved CR with IWG-2006 but not with overlap-MDS/MPN criteria because of persistent monocytosis in three of them and of persistent splenomegaly in the remaining three, leading to a lower CR rate of 13% with overlap-MDS/MPN criteria compared to 20% with IWG-2006 (*P*=0.03, binomial test). Two patients achieved OMR-CB with overlap-MDS/MPN criteria but only MR with IWG-2006 due to improvement of symptoms (arthralgia) for one and splenomegaly for the other one. Considering non-responders, nine patients were stable according to overlap-MDS/MPN but had progressive disease per IWG-2006 because of worsening of cytopenia. The more stringent definition of progression by overlap-MDS/MPN seems relevant, as six patients who had progressive disease per IWG-2006 at first assessment finally achieved response, whereas no patients with progression per overlap-MDS/MPN achieved response (Supplementary Information). Likewise, the more stringent definition of CR by overlap-MDS/MPN, which includes improvement of proliferative features, resulted in a lower CR rate (13%) compared to IWG-2006 (20%). These discrepancies will need to be considered when retrospectively comparing results from studies using different sets of response criteria, as well as for the design of future studies.

Median response duration was 22.3 months (IQR: 10.6–35.7 months) according to overlap-MDS/MPN criteria and 13 months (IQR: 5.8–22.3 months) according to IWG-2006. Paired survival analysis in the 45 patients with response according to both criteria confirmed the shorter duration of IWG-2006 defined responses (HR=2.83 (95% CI: 1.54–5.20), *P*=0.0008).

Considering response as a time-dependent variable and censoring at transplant, achievement of any IWG-2006 response (HR=0.42 (95% CI: 0.22–0.82) *P*=0.01) or of any overlap-MDS/MPN response (HR=0.34 (95% CI: 0.18–0.67), *P*=0.002) lead to prolonged OS. Both sets of response criteria led to similar predictive power for overall survival (Akaike Information Criterion [AIC] 310.0 and 312.6 for overall overlap-MDS/MPN and IWG-2006 criteria, respectively). When focusing on overlap-MDS/MPN response subtypes, achievement of CR had the strongest benefit on OS (HR=0.20 (95% CI: 0.07–0.60), *P*=0.004); achievement of OMR translated into significant OS benefit (HR=0.29 (95% CI: 0.13–0.66), *P*=0.003) whereas achievement of CB (or PMR in three cases) without OMR had limited impact on OS (HR=0.55 (95% CI: 0.24–1.26, *P*=0.16), Figure 1. The lack of OS improvement seen in patients with CB or PMR may be due to the small size of this group (*n*=20).

Eleven patients received ASCT, after a median of 6 cycles (IQR 3–13) of HMA, including 9 who had reached a response per overlap-MDS/MPN criteria (three OMR with CB, four OMR, two CB, including one CB-Spl), and only 4 of whom had reached a response per IWG-2006 criteria (3 HI and 1 mCR). One patient had spleen size reduction and was classified as stable disease per IWG-2006 criteria, and the remaining four patients were classified as progressive disease because of worsening cytopenias The small number of transplanted patients precludes analysis of post-transplant outcome, but these data suggest overlap-MDS/MPN responses encompass clinically meaningful improvement already considered for the decision to transplant in daily practice.

Finally, the impact on OS of dissociated responses was difficult to assess because of the small number of patients (*n*=11), achieving a response per overlap-MDS/MPN but not per IWG-2006 criteria, and because five of them subsequently received allogeneic transplantation.

In conclusion, we report the first retrospective validation of these new overlap-MDS/MPN criteria in the setting of CMML treated with HMA. By taking into account improvement of myeloproliferative features and by allowing to classify patients with dissociated responses more easily, not considering isolated increase in bone marrow blasts or worsening of cytopenia as a progression, overlap-MDS/MPN criteria increase response rates as well as response duration.

These criteria remain to be evaluated in prospective studies, with a comprehensive evaluation of clinical symptoms and systematic cytogenetic examinations, to confirm their contribution in defining robust short-term endpoints for future CMML clinical trials.

## Figures and Tables

**Figure 1:**
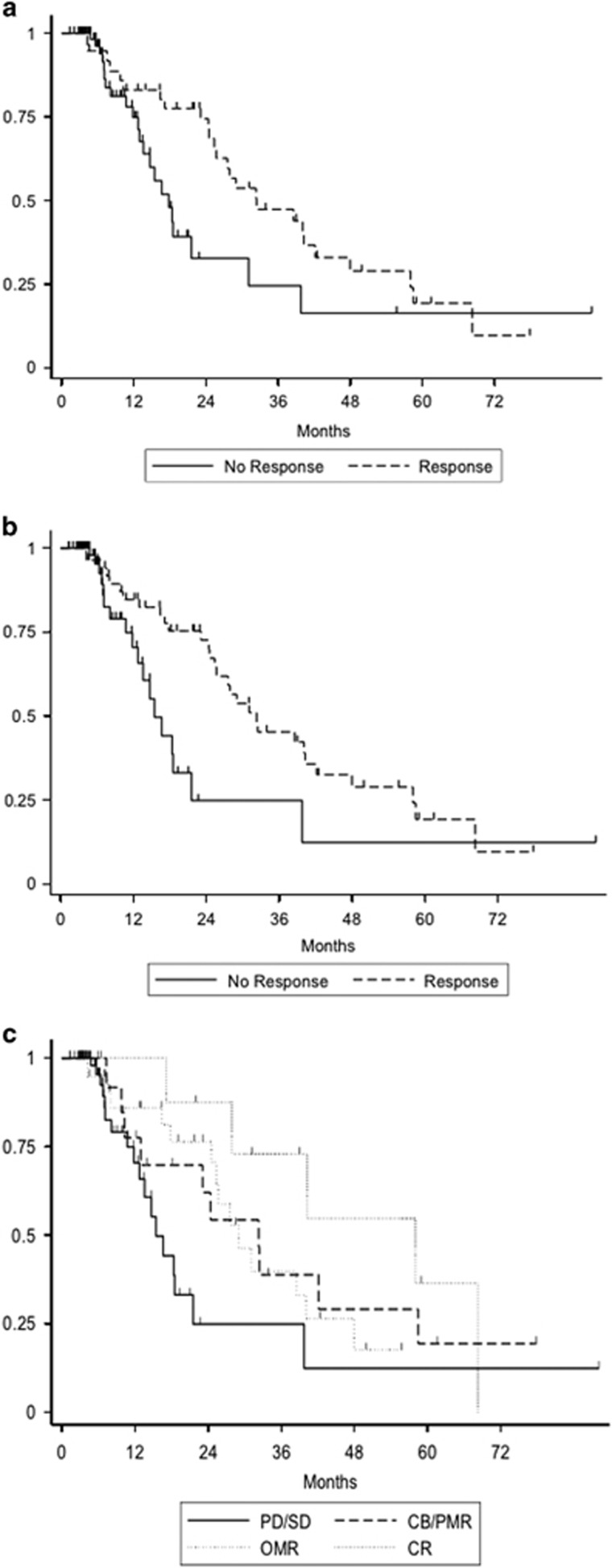
Simon–Makuch plot of overall survival (OS) according to the achievement of (**a**) any IWG-2006 defined response, (**b**) any overlap-MDS/MPN defined response and (**c**) type of overlap-MDS/MPN defined response: complete response (CR), optimal marrow response (OMR), clinical Benefit (CB) and partial marrow response (PMR), and lack of response (Stable disease (SD) or progressive disease (PD)). Achievement of response was considered as a time-dependent variable and OS is censored at the date of last follow-up or allogeneic transplantation.

**Table 1:** Concordance of best responses

	*Overlap-MDS/MPN criteria*					
	*Responders*			*Non-responders*		
	*No. of patients*	*IWG-2006*	*Overlap-MDS/MPN*	*No. of patients*	*IWG-2006*	*Overlap-MDS/MPN*
*IWG-2006 criteria*						
Responders	45 (57%)			0		
	10	CR	CR			
	10	mCR-HI	OMR-CB			
	6	mCR	OMR			
	11	SD-HI	SD-CB			
	1	CR	OMR-CB			
	1	CR	OMR			
	4	CR	SD-CB			
	2	mCR	OMR-CB			
Non-responders		11 (14%)			23 (29%)	
	1	SD	OMR	10	SD	SD
	1	SD	PMR	4	PD	PD
	3	SD	SD-CB	9	PD	SD
	1	PD	OMR-CB			
	4	PD	OMR			
	1	PD	SD-CB			

Abbreviations: CB, clinical benefit; CR, complete response; HI, haematologic improvement; mCR, marrow complete response; OMR, optimal marrow response; PD, progressive disease; PMR, partial marrow response; SD, stable disease.
