# Supplementary material for: Validation of response assessment according to international consortium for MDS/MPN criteria in chronic myelomonocytic leukemia treated with hypomethylating agents
Source: Blood Cancer J. 2017 May 12;7(5):e562–. doi: 10.1038/bcj.2017.41 (PMC5518885; doi:10.1038/bcj.2017.41)
Supplement: Supplementary Information [file bcj201741x1.docx]

**EXTENDED METHODS**

**Patients**

We updated clinical data from CMML patients treated by AZA or DAC included in GFM CMML clinical trials (EudraCT #2008-000470-21) {Braun, 2011 #2352} or registry (PHRC MAD-06) {Itzykson, 2013 #4157}. Patients with acute myeloid leukemia (AML) transformation before initiation of HMA were excluded. CMML diagnosis and stratification was made according to WHO 2008 criteria {Swerdlow, 2008 #1694}. Performance status was based on WHO scale. Splenomegaly was defined as enlarged spleen at clinical examination or imaging. Immature myeloid cells (IMC) were defined as peripheral blasts, promyelocytes, myelocytes and metamyelocytes. Bone marrow blast count included agranular blasts, myeloblasts and promonocytes {Goasguen, 2009 #2017}. Cytogenetic risk was assessed according to Such, 2011 #2910}. *ASXL1* exon 12 mutational status, determined by Sanger sequencing as previously described {Gelsi-Boyer, 2009 #2037}, was available for the majority of patients. Prognosis at initiation of HMA treatment was based on the CMML-specific prognostic scoring system (CPSS) {Such, 2013 #4283} and the GFM score {Itzykson, 2011 #2357}. Patients received AZA according to European Medicines agency’s label and DAC as part of the GFM CMML phase II study {Braun, 2011 #2352}, with standard schedules: AZA 75mg/m^2^/day on days 1-7 of each 28-day cycle, and DAC 20mg/m^2^/day on days 1-5 of each 28-day cycle.

**Responses**

***Response according to MDS-IWG***

Response, relapse and progression were defined according to Cheson et al. {Cheson, 2006 #116} Complete remission (CR) was defined as bone marrow blasts ≤ 5% together with hemoglobin (Hb) ≥ 11g/dl, platelets (Plt) ≥ 100 x 10^9^/L, absolute neutrophil count (ANC) ≥ 1 x 10^9^/L, and no peripheral blast. Partial remission (PR) was defined as all CR criteria except that bone marrow blasts decreased by 50% but were still ≥ 5%. Marrow complete response (mCR) was defined by bone marrow blasts ≤ 5% and decreased by ≥ 50% (only for patients with > 5% bone marrow blasts at initiation of HMA), with persistence of cytopenia (additional hematologic improvement was specified). Hematological Improvement (HI) included erythroid response (HI-E), for patients with pretreatment Hb < 11g/dL and an increase of Hb ≥ 1.5g/dL, or a reduction ≥ 4 units / 8 weeks in red blood cells (RBC) transfusions; platelet response (HI-P), for patients with pretreatment Plt < 100 x 10^9^/L and an absolute increase of ≥ 30 x 10^9^/L if pretreatment platelets were > 20 x 10^9^/L, or a 100% increase and > 20 x 10^9^/L if pretreatment platelets were < 20 x 10^9^/L; neutrophil response (HI-N), for patients with pretreatment ANC < 1 x 10^9^/L, as at least 100% increase and an absolute increase > 0.5 x 10^9^/L. Relapse for responding patients was defined as a return of the bone marrow blast percentage to its pre-treatment value, a decrement of ≥ 50% from maximum response levels in granulocytes or platelets, or a reduction in Hb by > 1.5g/L or appearance of a transfusion dependence. Progression was defined by bone marrow blasts increase ≥ 50%, at least 50% decrement from maximum response in granulocytes or platelets, or reduction in Hb by ≥ 2 g/dL, or transfusion dependency. Stable disease (SD) was defined as failure to achieve response without evidence of progression.

***Response according to overlap-MDS/MPN response criteria***

Response, relapse and progression were defined according to Savona et al {Savona, 2015 #4871}. CR was defined as bone marrow blasts ≤ 5%, WBC ≤ 10 x 10^9^/L, Hb ≥ 11 g/dL, Plt ≥ 100 x 10^9^/L, ANC ≥ 1 x 10^9^/L, peripheral blast 0%, IMC ≤ 2%, monocytes ≤ 1 x 10^9^/L, and complete resolution of extramedullary disease if initially present. Partial remission (PR) was defined as normalization of peripheral counts and hepatosplenomegaly, with a decrease by 50% of bone marrow blasts remaining > 5%. Optimal marrow response (OMR) was defined as marrow criteria for CR without normalization of peripheral blood count, if pretreatment marrow blast count was > 5%. Partial marrow response (PMR) was defined as marrow criteria for PR without normalization of peripheral counts. Clinical benefit (CB) was evaluated in patients without CR/PR or progression criteria and included erythroid benefit (CB-E) defined as increase in Hb ≥ 2 g/dL (if < 11g/dL at baseline) or transfusion independency (TI) for ≥ 8 weeks for patients requiring ≥ 4 RBC units per 8 weeks at baseline; platelet benefit (CB-P) defined as transfusion independence in patients requiring ≥ 4 platelets transfusions per 8 weeks at baseline, or an increase from ≤ 20 x 10^9^/L to > 20 x 10^9^/L and by at least 100% or increase of at least ≥ 30G/L if > 20 x 10^9^/L and ≤ 100 x 10^9^/L at baseline; Neutrophil benefit (CB-N) defined as 100% increase and absolute increase ≥ 0.5 x 10^9^/L if ANC pretreatment was ≤ 0.5 x 10^9^/L or 50% increase and an absolute increase ≥ 0.5 x 10^9^/L if ANC pretreatment was > 0.5 x 10^9^/L and ≤ 1 x 10^9^/L. Spleen benefit (CB-Spl) was defined as a significant reduction in spleen size based on palpation. Symptom benefit (CB-Sym) was defined as a significant improvement of clinical symptoms. Retrospective assessment of CB-Spl and CB-Sym was based on systematic retrospective chart review. Progression corresponded to assessment of a combination of 2 major criteria, or 1 major and 2 minor criteria or 3 minor criteria. Major criteria corresponded to significant increase in blast count (≥ 50%), evidence of cytogenetic evolution and worsening of splenomegaly or other extramedullary disease. Minor criteria included transfusion dependency, significant loss of best response or worsening from baseline of cytopenia (≥ 50% reduction in granulocytes or platelets count or reduction in Hb by ≥ 1.5g/dL), worsening symptoms or signs of clonal evolution.

**Statistical analyses**

Agreement between criteria was assessed by Cohen’s kappa, a measure of reproducibility ranging from 0 (no concordance) to 1 (perfect concordance) (Cohen et al. 1960). Overall Survival (OS) was defined as the interval between the HMA onset and death from any cause. AML-free survival (AMLFS) was defined as the time between the date of beginning of HMA and date of death or AML transformation according to WHO criteria (WHO 2008). When indicated, OS and AMLFS were censored at the date of last-follow up or transplant and Cox models were performed by Mantel-Byar method, considering achievement of response at first assessment as a time-dependent variable. Survival plots were realized according to Simon and Makuch. Response duration was evaluated censoring at last follow-up, death or transplant. Paired survival analysis of MDS-IWG and overlap-MDS/MPN response duration was performed by a Cox model with frailty. All time to event analyses were stratified on the hypomethylating agent received. All tests were two-sided, retaining p<0.05 as statistically significant. Statistical analyses were performed on STATA and R.
